# Defining and validating a multidimensional digital metric of health states in chronic back and leg pain

**DOI:** 10.1038/s41746-025-02084-1

**Published:** 2025-11-21

**Authors:** Jenna M. Reinen, Carla Agurto, Guillermo Cecchi, NAVITAS and ENVISION Studies Physician Author Group, Richard Rauck, Eric Loudermilk, Julio Paez, Louis Bojrab, John Noles, Todd Turley, Mohab Ibrahim, Amol Patwardhan, James Scowcroft, Rene Przkora, Nathan Miller, Gassan Chaiban, Boston Scientific Research Scientists Consortium, Dat Huynh, Kristen Lechleiter, Brad Hershey, Rex Woon, Matt McDonald, Jeffrey L. Rogers

**Affiliations:** 1 Digital Health, IBM Research, T. J. Watson Research Center, Yorktown Heights, NY USA; 2 The Center for Clinical Research, Winston Salem, NC USA; 3 PCPMG Clinical Research Unit, Anderson, SC USA; 4 South Lake Pain Institute, Clermont, FL USA; 5 Forest Health Medical Center, Ypsilanti, MI USA; 6 River Cities Interventional Pain Specialists, Shreveport, LA USA; 7 Hope Research Institute, Phoenix, AZ USA; 8 University of Arizona, Department of Anesthesiology, Tucson, AZ USA; 9 KC Pain Centers, Lee’s Summit, MO USA; 10 University of Florida, Gainesville, FL USA; 11 Coastal Pain and Spinal Diagnostics, San Diego, CA USA; 12 Ochsner Clinic Foundation, New Orleans, LA USA; 13 Data Research and Engineering, Boston Scientific, Valencia, CA USA; 14 Clinical Research, Boston Scientific, Valencia, CA USA

**Keywords:** Chronic pain, Scientific data, Quality of life

## Abstract

Chronic pain (CP) is a debilitating condition that extends beyond persistent pain, influenced by physiological and psychological factors. However, clinical trials often evaluate outcomes solely on self-reported pain amplitude. To address this, we aimed to derive a single metric from multidimensional digital data to comprehensively represent wellness in lower back and leg pain. Daily-reported data were collected for five years (>190 K samples, *n* = 498, from NCT01719055/NCT03240588), comprised of clinical assessments, digitally-reported symptoms, text responses, and smartwatch-based actigraphy. Clustering analysis of the digital data identified five novel symptom clusters. They were validated by comparing centroid distances to standard assessments, revealing five ordinal best-to-worst states (r = 0.34 to r = −0.51, ps < 0.001), even when pain magnitude was similar. Further, patients’ text messages about their status associated better with the clusters than pain reports alone. This solution extends beyond a recapitulation of pain level, yielding non-obvious, meaningful states that serve as an actionable metric in CP care.

## Introduction

At some point in their lives, at least 20% of Americans experience chronic pain (CP)^1,2^. It is not only associated with a significant impact on mental and physical health, employment, and social interaction, but the CP disease burden includes the opioid crisis. As a result, there is an urgent incentive to understand CP and to predict its onset and progression. Two complicating factors are its complexity and longitudinal nature. Initially, it is characterized primarily by prolonged pain ( > 3–6 months^3,4^) after an injury has healed^5^. Past the initial diagnosis, well-being and subjective pain reports are bidirectionally impacted by sleep, mobility, medication use, psychosocial factors, and other variables^6^. For example, sleep disturbances are a core factor in CP^7^, can worsen the experience of pain^8^, and contribute to its chronicity^9^. CP is also profoundly modulated by emotion and cognitive processing^10^, as CP diagnosis may be predicted by emotional states^11^ and related brain circuitry^12^. Despite this, known comorbid symptoms such as mood, attention, and sleep are not consistently monitored throughout the disease, even though they are known to affect outcomes meaningfully^13^. Thus, the multidimensional interaction of these associated symptoms is currently insufficiently understood when evaluating important clinical outcomes, and there remains a need to represent CP from a holistic perspective.

Many CP clinical trials use temporally coarse data collected at in-clinic visits that are often infrequent relative to the evolution of the pain, and report statistically derived relationships between a few variables. Technical advances in digital health can address these gaps by capturing meaningful symptoms as patients go about their daily lives^14–16^ at frequencies relevant to the evolution of the symptoms. Additionally, appropriate forms of artificial intelligence (AI) have shown promise beyond standard statistical approaches in identifying disease metrics because they can recognize patterns or joint representations in large, heterogeneous data streams. Machine learning has shown some success in CP when identifying pain^17^, distinguishing subgroups^18^, and improving diagnoses^19^, but it has only contributed minimally to identifying structure in biological and clinical data^20^ and in treating or managing pain directly^19,21^. Further, studies using combined multi-modal objective and subjective data (*e.g*., sensors *with* patient self-reports) to understand CP are broadly underdeveloped. Another issue is that for many clinicians who manage CP, predictive algorithms infrequently generate actionable results and lack information about clinical contextualization, labels, or other qualitative aspects. Considering this, we aimed to leverage modern AI (*i.e*., unsupervised clustering methods) to shed light on the complex symptom structure of CP across time in daily collected clinical trial data. We seek to understand better how groups of symptoms – including but not exclusive to pain – cluster together across time and how this connects to critical outcomes such as function and quality of life.

In this study, we conducted discussions with the Physicians Author Group and researchers intended to ensure the study design and results would facilitate the experience of interpreting longitudinal and raw time course data for clinicians, and to formulate outcome variables for prediction algorithms seeking to predict or optimize treatments. Our goal with this approach was to develop a computationally-informed metric that integrates multi-level symptom data instead of relying on pain reports alone. We aimed to create a single metric that is easily communicated, clinically relevant, and can be used in longitudinal assessments of CP, given the known interactive nature of types of symptoms across the course of the disease. To this end, we used data from a longitudinal, multi-center clinical trial across several years in hundreds of individuals treated for chronic lower back and leg pain using spinal cord stimulators (SCS). While chronic lower back and leg pain does not represent all types of CP, it notably does constitute the most common types of reported pain locations^1^. We included multiple symptoms expected to impact the quality of life in this cohort and in CP individuals more generally, including mood, sleep, medication use, alertness, and activity, and combined them with an unsupervised clustering method. In a subsequent analysis, we added watch-based actigraphy measures to quantify activity intensity in this model. To validate the results, we compared clustering characteristics to periodic assessments of function (disability), quality of life, and open-text self-report responses from the participating patients, contextualizing the unsupervised output. We will discuss the implications of these findings and weigh the advantages of using a multidimensional health metric in CP instead of relying upon pain magnitude alone, as well as the broader applications of this methodological approach.

## Results

### Data characteristics and study details

**Fig. 1:**
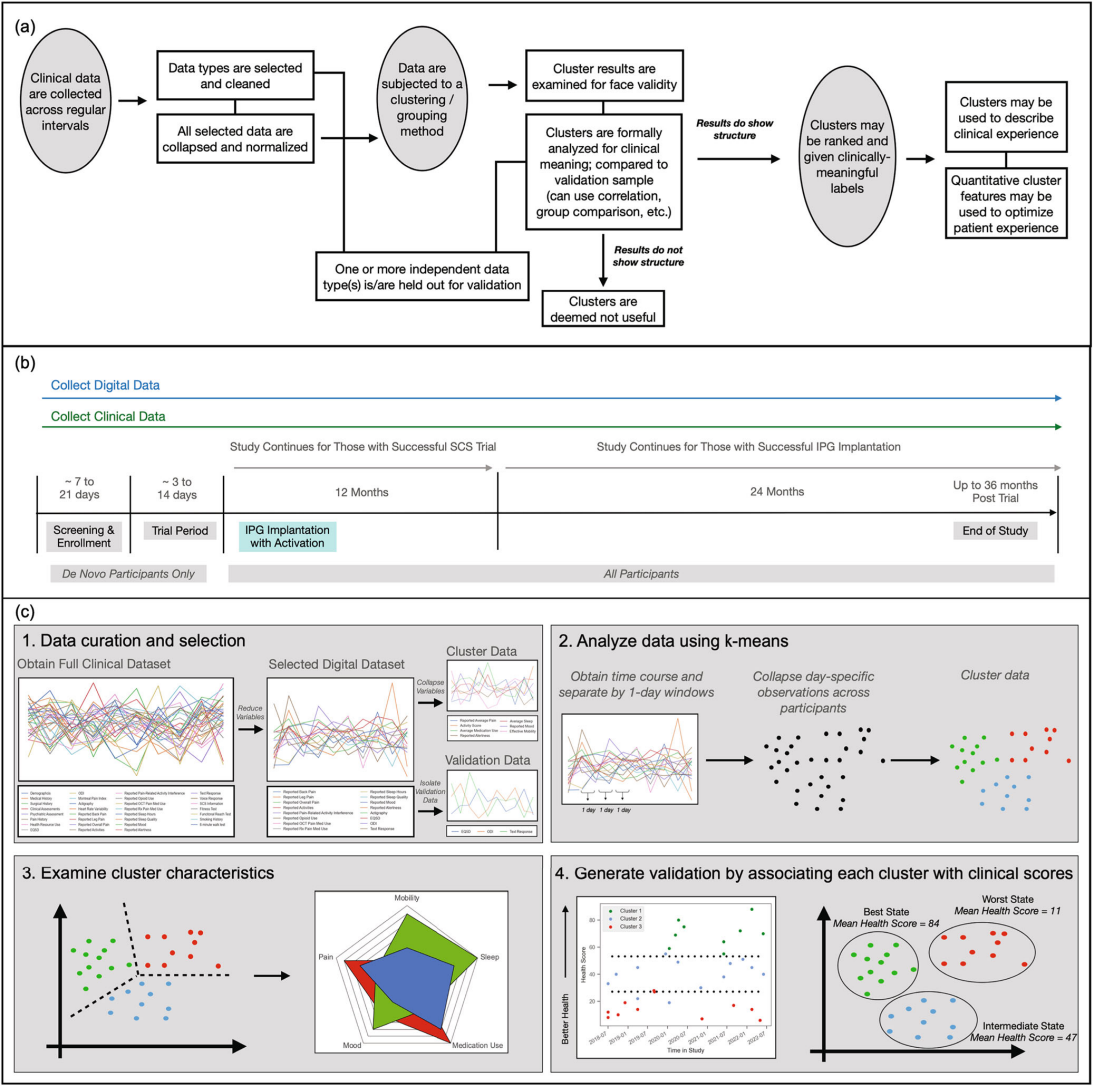
Schematic of Clinical Trial and Hypothetical Analysis Overview. (**a**, Upper panel) This schematic describes the general approach that was developed to obtain and curate longitudinal clinical data, subject it to a grouping algorithm, and explore whether the output was significantly aligned with clinically meaningful, held-out data. (**b**, Middle panel) In the present study, eligible neurostimulation-naive participants were recruited from pain clinics as de novo participants. Additionally, participants who were already enrolled in a prior trial and had a successful spinal cord stimulator (SCS) trial were also offered participation in the study after completing a baseline visit. Following screening and enrollment, participants had a trial procedure visit, which trained them on the usage of the device, and pain relief was assessed. Those with a successful trial received an implantable pulse generator (IPG) implantation and were followed for as long as 36 months. (**c**, Lower panel) (1) Data from (B) were applied to the framework in (A) in that they were curated from a large clinical trial dataset ( > 190 K samples) and were selected for hypothetical contribution to wellness in chronic pain patients, availability, and other factors. (2) Data were subjected to a k-means clustering analysis, and (3) results were inspected. (4) The clusters were validated by comparing the cluster properties to the health assessments (disability and quality-of-life). Finally, a label and rank were assigned to the clusters.

The conceptual framework (Fig. 1a) was implemented using data from an observational clinical trial that recruited individuals diagnosed with intractable neuropathic pain seeking SCS therapy (see Fig. 1b, Methods, and Supplementary Materials). The entire dataset included 190,580 samples from 498 participants, starting in August 2017. After applying the data inclusion criteria, which was intended to ensure ample, quality data for the daily digital responses for mood, sleep, pain, alertness, medication use, and activity, the clustering analysis using only questionnaire data included 375 participants with 50,620 complete samples (all 14 entries per day were available) collected between May 22, 2018, and October 26, 2022, with a median of 412 ( + /−349) days per person. For the clustering analysis that included actigraphy (mobility) data, 327 participants across 30,086 samples were included. The validation data underwent a similar process to ensure that they were temporally aligned to the cluster data. This analysis, which aimed to verify the unsupervised clustering results, included 1031 samples from 332 participants also from May 2018 to October 2022, indicating that the assessments successfully spanned a similar chronology to the data used to calculate the clusters. The analysis that included actigraphy data used a validation sample of 647 with 261 participants, also within the same range of time. Additional details about the patient cohort are found in Table 1.

**Table 1:** Participant demographics and characteristics

	Category	Values: No actigraphy	Values: With actigraphy
Demographics	Number of subjects	375	327
	Sex	38.7% male (145)	37.6% male (123)
	Age (years)	60.2 + /− 12	60.0 + /− 11.9
	Race	Caucasian: 317, Black: 29, Hispanic: 18, American Indian: 5, Other: 6	Caucasian: 273, Black: 29, Hispanic:16, American Indian: 5, Other: 4
Pain history	Years since onset of pain	16.2 + /− 13.4 years	16.2 + /− 13.2
	Pain location	Low back pain: 96.3% Unilateral lower extremity: 32.5% Bilateral lower extremity: 58.7%	Low back pain: 95.7% Unilateral lower extremity: 33.3% Bilateral lower extremity: 58.4%
	Implanted with SCS	72.27%	76.45%
	Diagnosis being treated by SCS	Lumbosacral Radiculopathy: 61.1% Failed Back Surgery Syndrome: 49.9% Other: 47.5%	Lumbosacral Radiculopathy: 61.5% Failed Back Surgery Syndrome: 49.5% Other: 44.6%
	Insurance coverage	Medicare: 174 Private insurance: 148 Medicaid: 14 Worker’s compensation:13 Other: 26	Medicare: 155 Private Insurance: 127 Medicaid: 12 Worker’s compensation: 10 Other: 23
	Concomitant therapies	OTC meds: 83.2% Chronic opioids: 71.7% Prescription non-opioids: 82.7% Complementary and Alternative Therapy: 49.6% Physical therapy: 79.2% TENS unit: 53.6% CBT: 14.7%	OTC Meds: 84.7% Chronic opioids: 72.8% Prescription non-opioids: 83.8% Complementary and Alternative therapy: 48.6% Physical therapy: 80.1% TENS unit: 54.1% CBT: 14.7%
Study data	Follow-up duration (days)	515.3 + /− 492.3	518.0 + /− 478.6
	% of Data with SCS	78.8%	79.12%
	Days of data for study period	419.1 + /− 384.7	391.5 + /− 377.6

The present table depicts the sample demographic and clinical characteristics for (middle column) the sample with only questionnaires and no actigraphy, and (right column) for the sample with both questionnaires and actigraphy.

### Clustering results and validation

**Fig. 2:**
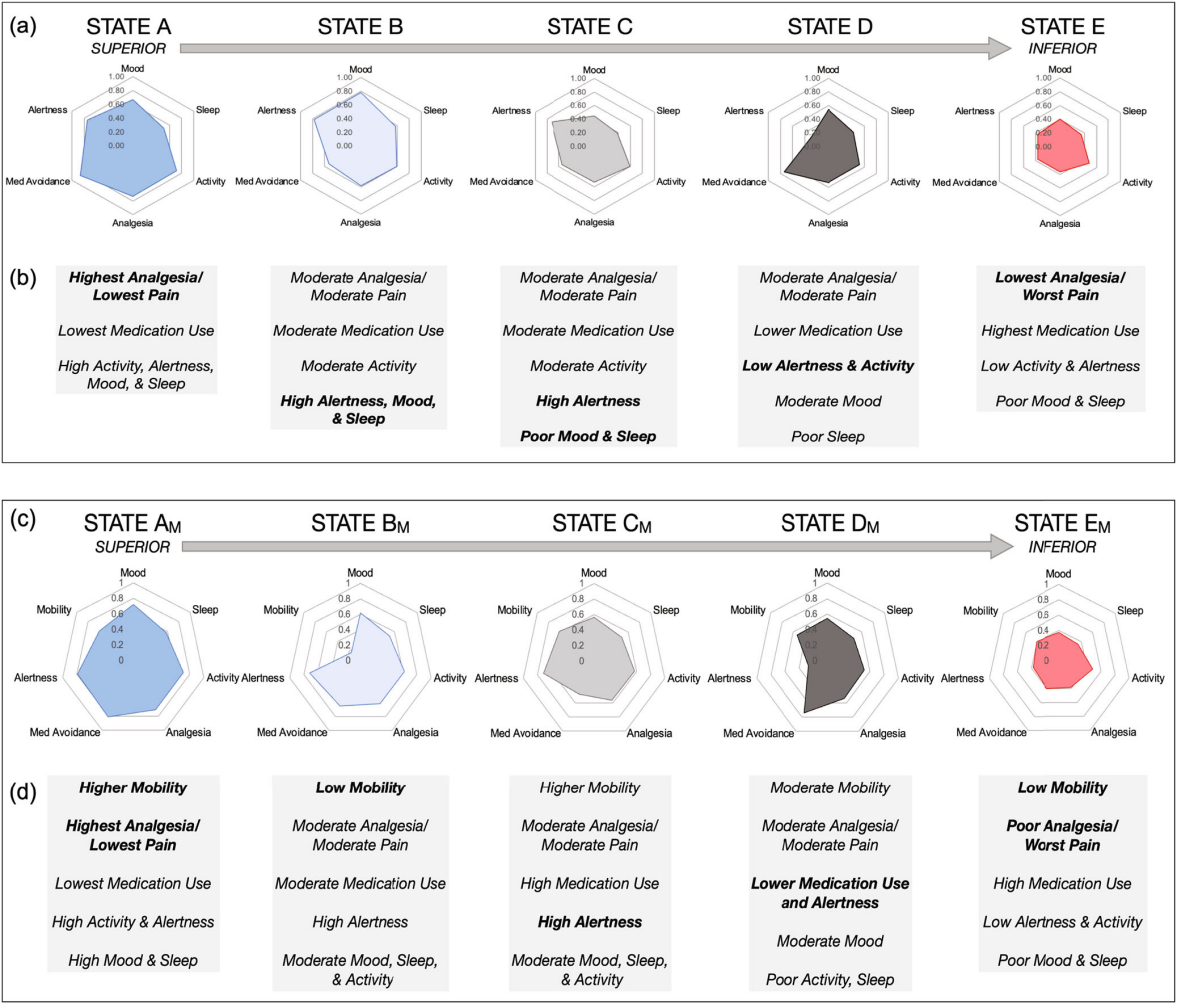
K-means results for a 5-cluster solution. The clustering results derived from (**a**, upper panel) questionnaires alone (*n* = 375 across 50,624 samples) revealed five states that were stratified on a negative-to-positive spectrum based on reported health symptoms consisting of mood, sleep, activity, pain, medication use, and alertness (here, inverse values are taken for pain and medication so that all features may visualized on the same good-to-bad scale). **b** Qualitative interpretation indicates that the best state (State A) demonstrates a high mean of mood, sleep, activity, alertness, analgesia (1 - reported pain), and medication avoidance (1 - medication use). Conversely, the worst state (State E) shows the opposite pattern. This analysis was repeated with actigraphy-based effective mobility (**c**, lower panel) for both questionnaires and actigraphy (*n* = 327 participants across 30,086 samples). **d** Similarly, a qualitative interpretation and comparison across states is provided for this model. For a direct visual comparison, see Supplementary Fig. 5.

Following data curation, a k-means clustering analysis was implemented to determine whether clinically meaningful clusters could be derived from the daily digital data (Fig. 1c). The clustering results identified a range of two to five (k = 2 to 5) stable clusters. Given prior findings that supported the presence of five states in CP^22^ and the value of increased granularity with additional clusters, we chose to focus on the five cluster solution. Feature characteristics for the five clusters were examined by first inspecting mean feature values associated with each cluster produced by the model (Fig. 2a). A clear pattern emerged in which desirable characteristics (*i.e*., better reported mood, sleep, activity, and alertness, and lower reported pain and medication use) were associated in the best cluster, or *superior state*, and undesirable characteristics clustered into the worst cluster, or *inferior state*. Intermediate clusters appeared to represent incremental steps between the superior and inferior states but notably included near-identical pain magnitude levels. Several features, including alertness and medication use, differentiated between them. The cluster model also showed consistency across periods of time and response rates (see Supplementary Figs. 2-3).

**Fig. 3:**
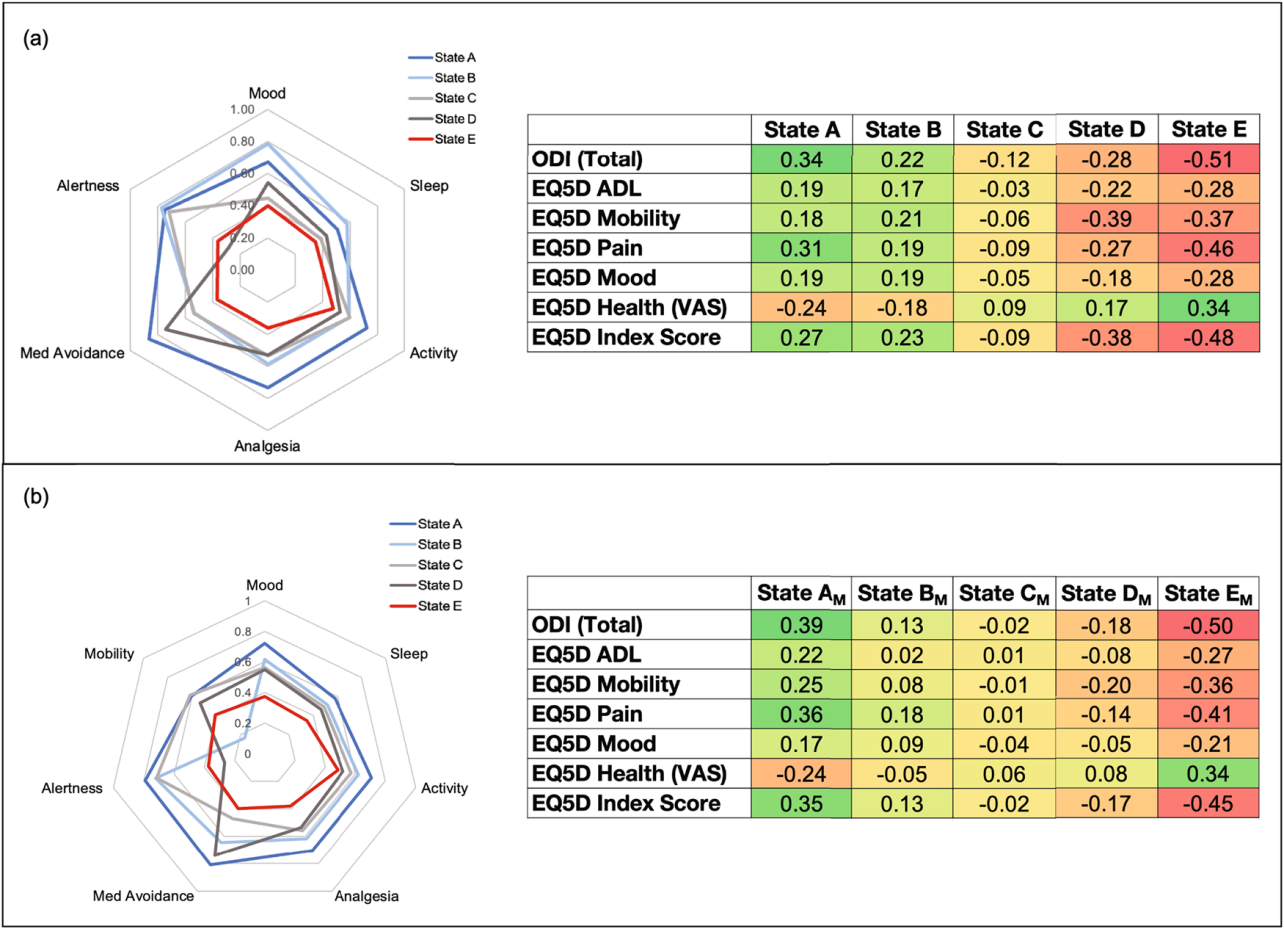
Validation using independent clinical metrics in a questionnaire sample. (**a**, Upper panel) To validate clustering results, we compared independently collected standard assessments (Oswestry Disability Index, or ODI, and the Euro Quality of Life metric, or EQ5D) by calculating the correlation between ODI and EQ5D with the cluster centroid distances for each state, within a week of each other. In both the primary model and the model that included both questionnaires and effective mobility, the best state (State A) was characterized by longer centroid distances and higher disability and pain scores, meaning the states with poor health measures were “far” from this state. Conversely, the worst state (State E) was characterized by short distances correlating with high disability and pain scores. Both solutions indicated a clear ranked order across the five states. (**b**, Lower panel) This process was repeated with mobility (actigraphy) data, and a similar pattern emerged (M denotes “mobility” in the state labels).

In order to confirm the cluster ranking as best-to-worst, a validation analysis was implemented to compare the clusters to held-out data comprised of disability (Oswestry Disability Index, or ODI^23^) and quality of life scores (the 5-factor Euro Quality of Life, or QoL^24^). Here, the validation scores were aligned with the cluster assignments by date, and correlations were calculated between the validation scores and cluster similarity characteristics (centroids). Indeed, the superior state was associated with lower disability and better quality of life; the opposite was true for the inferior state. Specifically, the results from the correlation analysis between the disability score and the cluster centroids indicated longer (further away) centroid distances for the superior state correlated significantly in a positive direction with the ODI or associated with high ODI scores. In other words, dissimilarity with the best state was associated with higher ODI values (severe disability), and similarity with the best state was associated with lower ODI values (lower disability). Conversely, shorter (closer) centroid distances are associated with higher ODI values for the inferior state, indicating cluster proximity or similarity associated with worse disability scores. The intermediate cluster centroids correlated with ODI values in a distinct order, suggesting that each associated with ODI in an ordinal way. A similar pattern and rank were observed for the QoL values in each sub-score. Notably, the EQ5D-VAS-Health metric, which, unlike ODI, represents positive aspects of health, showed an inverse relationship relative to the prior findings (Fig. 3), further verifying the best-to-worst rankings.

**Fig. 4:**
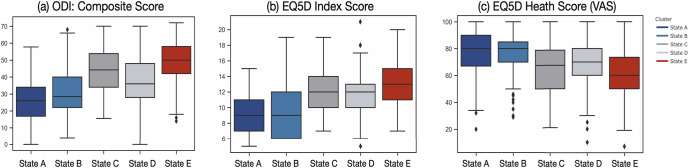
Relationships between states and clinical outcomes. The box plot depicts mean (line) and quartile values associated with each state; the whiskers denote the distribution, and points represent outliers. **a** The total score for the Oswestry Disability Index (ODI) and the (**b**) EQ5D (QoL) Index Score indicate that lower values(better outcomes) are associated with State A, and higher values (worse outcomes) are associated with State E. In contrast, (**c**) the EQ5D Health Score, for which higher values are associated with better outcomes, shows the opposite trend. These outcomes were chosen for their continuous nature and ability to compare direction and stratification. Repeated measures ANOVA tests showed significant differences in scores across clusters for all score types (*p* < 0.0001), with post-hoc tests confirming significant differences across clusters for all ODI scores and most EQ5D scores (ps<0.01), except States A vs. B (*p* = 0.41) and C vs. D (*p* = 0.31) for the Index Score, and for States A vs. B (*p* = 0.9) and C vs. E (*p* = 0.054) for the Health Score (VAS).

**Fig. 5:**
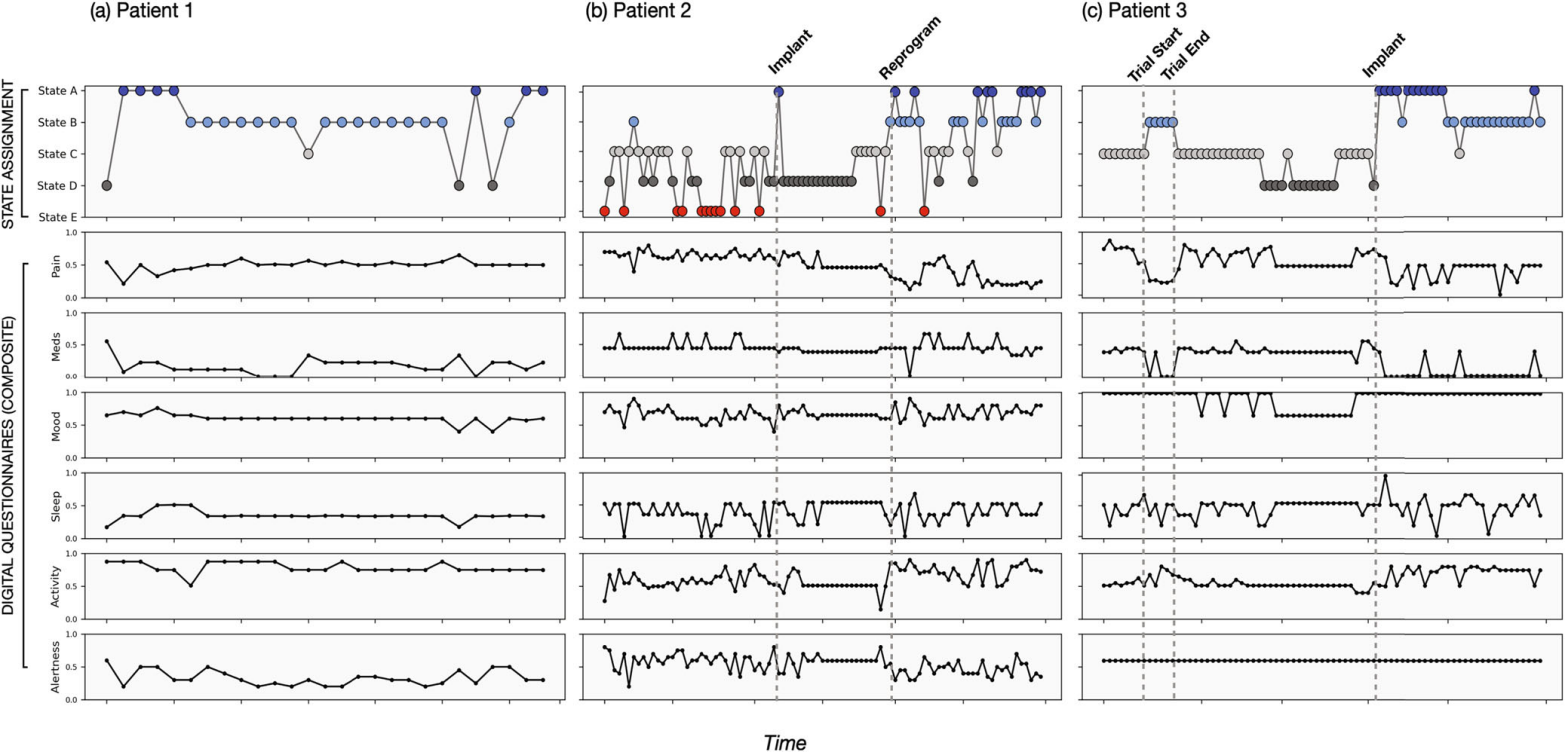
Patient data may be reduced to a single metric using Pain Patient States. Pain Patient States are expressed as a single variable representing many aspects of patient experience, including alertness, sleep, medication use, mobility, activities, mood, and pain magnitude. Above represent three real patient anecdotes depicting how states may reflect SCS-related clinical events: **a** an example of an individual who experiences moderate pain but remains in a good state; **b** a patient who shows state-based responses at implant and reprogramming; and **c** a patient who shows state-based responses during trial and implantation.

Given these findings, we assigned the clusters to distinct health states named A-E, with A being the superior state and E being the inferior state. Figure 2 presents a qualitative interpretation and comparison of the states. We also explored descriptive statistics for the standard assessments across the states given the assigned rank (Fig. 4) and anecdotal accounts for which the states represented episodic medical events during SCS treatment (Fig. 5). Adding actigraphy to the cluster model showed broad consistency with the questionnaires-only model. The qualitative clustering results (Fig. 2b) appeared to have similar feature characteristics and provided added insight that while high mobility is generally associated with the superior state (A_M_), low mobility may also be associated with a good state (B_M_), supporting prior findings that the association between pain and mobility is nontrivial. Also, a clear ranking was identified for this solution, with A_M_-E_M_ denoting the model that includes mobility (see Fig. 3b).

Finally, we examined the relationship between participants’ open-ended responses via text message to questions about their health, pain, day, and other topics (see Supplementary Table 1) and States A through E. The text responses were analyzed by calculating semantic similarity scores between the participant responses and positive and negative statements about well-being (see Supplementary Table 2). This calculation was used to create the text health score. Like the disability and QoL scores, the content of patients’ text messages also revealed that the clusters could be organized into ranked health states. Using a similar validation analysis, we found that the text health scores, for which positive values indicated better health, correlated significantly with the distance from the best state in the questionnaire-only model (r = −0.23, *p* < 0.000001, with a 95% confidence interval of −0.25 to −0.20; see Supplementary Table 3). The negative r values indicated that higher (better) text health scores correlated with shorter distance values in (more similar to) the best state. As a comparison, we examined the relationship between text health score alone and average daily pain report, which also correlated significantly in the same direction, in that higher pain and the text health score were inversely correlated (r = −0.14, *p* < 0.001, with a 95% confidence interval of −0.16 to −0.11). Using the model that included mobility, we again found a relationship between the text health scores and the best state (r = −0.28, *p* < 0.00001 with a 95% confidence interval of −0.31 to −0.25), revealing a likely higher r-value compared to the relationship between text health scores and pain (r = -0.16 with a 95% confidence interval of −0.19 to −0.13). Though the text responses varied in the extent and precision to which each participant adequately evaluated their well-being, the relationship between pain and states compared to the text health score was significant. However, it is notable that the range of the confidence intervals suggests that the states co-vary better with the text health score responses than with the magnitude of pain, implying that the states can authentically capture general well-being assessments at least as well, if not better than pain alone.

## Discussion

Using longitudinal data from hundreds of individuals experiencing CP across nearly five years, we created a validated, data-driven, multidimensional metric of well-being in CP, accounting for pain magnitude augmented by sleep, mood, alertness, medication, activities, and mobility. By integrating these features, which were assessed daily, we identified five symptom clusters, which we call Pain Patient States. The model’s output revealed a clear distinction between superior and inferior clusters, in which positive and negative symptoms were grouped at opposite ends of the spectrum. To validate the model, we compared centroid distances to assessments of disability, QoL, and open-form text self-reports. This analysis showed that the clusters held an ordinal property, in which the superior cluster was associated with lowest disability, highest QoL, and most positive text responses; the opposite pattern was shown in the inferior cluster. Critically, three intermediate clusters were ranked distinctly as they related to health but were remarkably similar in pain severity level. This suggests these clusters provide meaningful information about multidimensional CP health states beyond pain alone. We argue that the metric presented here tracks well-established wellness measures, may be remotely obtained, is both data- and expert-driven, and can be reduced to an interpretable measure that is informative for medical decision-making and outcomes prediction.

An advantage to this clustering approach is the ability to observe the intricate dynamics of pain-related symptoms, which, by extension, provides insights into biological mechanisms and clinical care. With this in mind, we chose a five-cluster solution that best balanced relative stability, improved granularity, and provided an understanding of patient experience. In prior analyses using a smaller sample^22^, more conservative cluster solutions of k = 2 to 3 were considered. While it is known that cluster instability increases with additional k, having too few clusters also limits the amount of information that may be inferred from a dataset. Using the cluster solution with maximal stable clusters allows one to understand symptom groupings and represents health status with better precision. Here, while it may be intuitive that high pain and poor mood co-occur, this approach allowed us to discover clusters in the Pain Patient States that were non-obvious. For example, states C vs. D showed differentiation based on reported alertness, which may be related to medication use and/or attentiveness and has been shown to modulate pain-related neural processing and behavior substantially^10,25^. We found two “good” states (States A_M_ and B_M_) associated with very different levels of mobility, supporting theories that the relationship between pain and movement is complex^26,27^, or potentially non-linear^28,29^. In the intermediate states, we observed similar reported pain magnitude. However, other features appeared to differentiate the states, such as high mood level for state B_M_ and low mood level for state C_M_. State D shows some degree of similarity to State C. Still, State D is associated with poor alertness, and State C is not, which may drive the association with poorer outcomes in State D. Considering these findings, we believe this approach reveals novel information about CP experience beyond measuring pain alone.

State characteristics covaried with widely-used health measures in a distinct, ordinal fashion. Comparisons with disability (ODI) and QoL (EQ5D) were used to provide clinical contextualization for our data-driven analysis, a step we consider critical when working with machine learning approaches that do not necessarily provide interpretation in the analysis output. However, while these standard assessments allowed us to contextualize and rank the states, these measures (ODI, EQ5D) are only referential measurements, and our main goal is not to replicate them. Instead, we aimed to develop a metric that offers value beyond these informative but separate assessments. Using a clustering approach instead of a composite measure affords the user a degree of flexibility when choosing input and validation variables and can generate proximity metrics like centroid distance, which indicates how similar an individual’s experience is to a good or bad state. This facilitates communication for clinicians and caregivers, enabling decision-making using this more straightforward, clinically useful metric.

We also validated the states using NLP-derived text health scores with the open-form text messages submitted by the participants, intended to capture a general self-assessment of their well-being. We found that the superior state centroids correlated similarly to the standard assessments. While pain also correlated with the text health score, the magnitude of the r value was not as high (r = −0.23 vs. r = −0.14), and 95% CI values did not overlap. This corroborates our previous findings that a multidimensional metric may capture a patient’s assessment of their overall well-being better than a singular value and that pain alone may not be sufficient. This analysis did have some limitations, as is customary in free-form responses, mainly in that all text messages were considered and not excluded for short or low-information responses (e.g., “Same,” “Fine,” “No,” etc.). Still, we could derive a significant relationship between pain and pain patient states in terms of the text health score. We interpret this to confirm that the Pain Patient States includes information about a multitude of symptoms and contains some signal in representing a patient’s opinion and voice about their clinical status.

We observed that actigraphy data, an objective measure not typically included in standard health assessments, fundamentally differs from self-reported questionnaires. This was evident given that actigraphy data only sometimes covaried with the activity score (e.g., states B and C have similar activity levels but not mobility). Unlike a questionnaire, actigraphy is challenging to interpret in its raw format, but it can offer unique, objective information about patient behavior, as shown in other disease trials. We have demonstrated that we could preprocess, quantify, and select features that may be incorporated into a multidimensional metric and that it may offer insight relative to a subjective metric alone, as evidenced by the higher correlation r-values corresponding to the superior state in the validation analysis.

To our knowledge, Pain Patient States are the first approach to derive a holistic CP metric from an unsupervised, data-driven clustering approach, which was validated with standard health assessments. This demonstrates that despite its data-driven nature, this model can be clinically contextualized and reveal non-obvious, valuable information about how symptoms group together in hundreds of individuals experiencing CP. Further, we collapsed both subjective (questionnaires) and objective (actigraphy) information, satisfying the need to represent a patient experience in multiple domains. We also identified a convergence of findings that open-form text responses and standard assessments showed an ordinal property in the states, regardless of pain level. As of yet, this approach has been implemented in two tangible ways. First, we integrated patient state assignment as a summary measure used to communicate chronic pain status within an Application Programming Interface (API) intended to manage, secure, analyze, and communicate health information^30,31^. Though many clinicians are familiar with interpreting patient data, this approach helps to visualize a comprehensive set of information on a dashboard, rather than to interpret multiple, longitudinal, and raw sensor-based datasets. This is an especially valuable feature when monitoring patients’ progression across long treatment periods. Second, a patient state value has been utilized to represent a single-value outcome within a reinforcement learning algorithm where a closed-loop health recommender algorithm aimed to optimize patient monitoring and outcomes^32^. Together, this demonstrates that patient states may be used to successfully and succinctly represent patient status within a digital health ecosystem, and in complex calculations that benefit from using a single value as an outcome variable.

Future work related to this approach involves several important considerations. Though chronic lower back and leg pain are the most common subtypes of CP^1^, this type of pain is characteristically distinct from CP conditions like migraine or fibromyalgia. It is also notable that this cohort specifically included individuals experiencing pain severe enough to warrant eligibility for SCS therapy. For these reasons, the clustering models developed here may not generalize to all forms of CP. However, we were able to reproduce a similar five-state model even after removing pain ratings (Supplementary Fig. 10), demonstrating that the overall structure of the clusters does not depend solely on pain, and may not rely on pain type. Consequentially, this *method* may be adapted to produce clustering models for other pain subtypes, particularly because most CP conditions are affected by the features involved in the model such as pain magnitude, sleep, medication use, activity, mobility, arousal, and mood. This can be achieved so long as the data includes 1) longitudinal, digital measures suitable to calculate clusters; and 2) measures that can serve as validation data (see Fig. 1a), neither of which are restricted to the specific inputs, questionnaires, or assessments used in this study. While the properties of such clusters may vary from the present work, their clinical meaning can be contextualized given the validation data, and may be further interpreted using domain knowledge. Additionally, several limitations should be addressed. This cohort was American, predominantly female, and Caucasian, and the participants suffered from specific pain-related etiologies. Future studies should aim to increase the size of longitudinal datasets, and include a more diverse set of participants to explore these states. Further, measures of social health and a more detailed analysis of medication use may add valuable dimension to the Pain Patient States. Like all data-driven approaches, these findings should be replicated in independent datasets. Still, Pain Patient States hold substantive promise in harnessing AI-inspired solutions to augment healthcare in CP.

## Methods

### Participants and data

Participants (Table 1) with chronic leg and back pain were recruited over the course of seven years from three, multi-center, prospective Boston Scientific-sponsored spinal cord stimulator (SCS, Boston Scientific, Valencia, CA) studies at up to 30 U.S. sites. RELIEF (Clinicaltrials.gov ID: NCT01719055, registration submitted October, 30, 2012) is a single arm, observational registry trial intended to understand approved neurostimulation practices for pain in clinics^33^. NAVITAS and ENVISION (both registered under Clinicaltrials.gov ID: NCT03240588, registration submitted July 11, 2017; see Fig. 1) aimed to characterize the relationship between objective measurements of pain and clinical outcomes in chronic pain patients seeking and receiving neurostimulation treatment. Inclusion criteria required that participants intended to receive or had received the SCS trial or implant, were at least 18 years old, and had been diagnosed with intractable neuropathic pain. The study was approved by the Western Institutional Review Board (WIRB), and informed consent was obtained before any study procedures. These studies collected a variety of self-reported, psychological, physiological, and other measures across the course of treatment via a custom digital health ecosystem^34,35^.

This broad set of clinical trial data included information about clinical visits, demographics, medical, pain, and psychiatric history, daily smartphone questionnaires, actigraphy, open-form text responses, health resource utilization, standardized pain-related assessments (e.g., for catastrophizing, fear-avoidance, quality of life, work productivity, disability, etc.), pain drawings, actigraphy, heart rate data and variability, daily self-reported health ratings, voice and text responses, fitness tests and other measures of activity, sleep assessments, actigraphy and mobility features, information about the spinal cord stimulator (SCS) sensor and programming, and other related variables. The reduced, curated set of data used in the present analysis was collected over four years and five months and chosen to reflect aspects hypothesized to primarily impact well-being in the lives of those with CP based on prior findings. This was comprised of 1) clinical data—including demographics, pain history, and standardized assessments (described below), and 2) digital data—including questionnaires, smartwatch actigraphy, and text responses collected via a smartphone-based app. The variables *not* chosen were either designated as exploratory variables that did not have a literature-established impact on pain, were previously deemed unrelated to outcomes of interest, or were insufficient in sample size.

### Data selection and preprocessing

The clinical trial is described in Fig. 1. Digital data was collected from each individual across the course prior to and during their treatment (or just during treatment for existing patients) with SCS therapy, and up to 36 months following implantation. Daily, self-reported questionnaires were recorded which reflected participants’ assessments of their own pain magnitude, mood, sleep hours and quality, alertness, medication use (opioid, prescribed non-opioid, and over-the-counter pain medication), and activity, including activities of daily living and pain-related activity interference (Table 2).

**Table 2:** Questions used in the subjective digital profile

Category	Question	Answer	Type
Mood	Please rate your mood right now (where 1 star is worst mood, 3 stars is neutral mood, and 5 stars is best mood)	Stars (1–5)	Single Select
Pain	Which number best describes the intensity of your overall pain? (0 for no pain and 10 for worst pain imaginable)	Input via a sliding scale between 0–10.	Number Slider
Pain	Which number best describes the intensity of your leg pain? (0 for no pain and 10 for worst pain imaginable, please select 0 if you don’t have any leg pain)	Input via a sliding scale between 0–10.	Number Slider
Pain	Which number best describes the intensity of your leg pain that your neurostimulator is programmed to treat? (0 for no pain and 10 for worst pain imaginable, please select 0 if you don’t have any leg pain)	Input via a sliding scale between 0–10.	Number Slider
Pain	Which number best describes the intensity of your low back pain? (0 for no pain and 10 for worst pain imaginable, please select 0 if you don’t have any low back pain)	Input via a sliding scale between 0–10.	Number Slider
Pain	Which number best describes the intensity of your low back pain that your neurostimulator is programmed to treat? (0 for no pain and 10 for worst pain imaginable, please select 0 if you don’t have any low back pain)	Input via a sliding scale between 0–10.	Number Slider
Sleep	How many hours did you sleep in the last day?	0–24	Number Slider
Sleep	Please rate the quality of your sleep where 1 star is poor sleep and 5 stars is great sleep.	Star (1–5)	Single Select
Activity	Does your pain interfere with your activities?	- I have no problems doing my usual activities / - I have slight problems doing my usual activities / - I have some problems doing my usual activities / - I have severe problems doing my usual activities / - I am unable to do my usual activities	Single Select
Activity	What type of activities did you do today? (Select more than one if applicable)	- Standing / - Sitting / - Housework / - Walking / - Running / -Dressing / -Bathing / -Feeding/eating / -Driving / -Cooking / -None of the above	Multi Select
Alertness	How rested, refreshed and restored do you feel on waking?	- Not at all / - Slightly / - Moderately / - Quite a bit / - Extremely	Single Select
Medication	Did you need to take prescribed pain medication (other than opioids) for your pain today? Examples may include: Neurontin/Gabapentin, Lyrica/Pregabalin, Cymbalta/Duloxetine, Voltaren Gel, Amitriptyline/Elavil	- No, I didn’t need to use any / - Yes, I needed to use less than usual / - Yes, I needed about the same amount / - Yes, I needed to use more than usual / - No, I do not have any pain medication (other than opioids) prescribed / - Prefer not to answer / - I don’t remember, unsure / - Other	Single Select
Medication	Did you need to take prescribed opioid medication for your pain today? Examples of opioids include: Morphine, Fentanyl, Oxycodone, Percocet, Hydrocodone, Vicodin	- No, I didn’t need to use any / - Yes, I needed to use less than usual / - Yes, I needed about the same amount / - Yes, I needed to use more than usual / - No, I do not have any opioid medications prescribed / - Prefer not to answer / - I don’t remember, unsure / - Other	Single Select
Medication	Did you need to take over-the counter pain medication today? Examples include: NSAIDs, Tylenol, Advil, Celebrex, Aleve, Ibuprofen, Capsaicin cream/gel	- No, I didn’t need to use any / - Yes, I needed to use less than usual / - Yes, I needed about the same amount / - Yes, I needed to use more than usual / - No, I do not have any opioid medications prescribed / - Prefer not to answer / - I don’t remember, unsure / - Other	Single Select

Subjective questions assessing mood, pain, sleep, activity, and medication were used to compute a subjective digital profile for each person in a day.

Each day, participants were asked to use this digital ecosystem on their phones to respond at least once, or up to twice, to the self-report questions. Participants were asked to wear a smartwatch, though doing so was an optional portion of the study. This was intended to assess mobility using accelerometer data from Galaxy Watch S2 and S3 (Samsung, Menlo Park, CA, USA) and the Garmin Venu 2S, (Olathe, KS) with custom application from Boston Scientific (Valencia, CA) to objectively assess mobility using accelerometer data. Actigraphy was collected daily at a sampling rate of 30–50 Hz, depending on the watch type used. Raw data was used to calculate effective mobility^22,36^, a novel metric of physical activity calculation based on duration and intensity, and categorized from Zone 0 (least active) to Zone 4 (most active), which is described below in additional detail.

To validate the results of the clustering analysis, we used clinically validated assessments (Oswestry Disability Index^23^, or ODI, and the 5-factor Euro Quality of Life, or EQ5D QoL^24^) and open-form text responses. This choice aimed to utilize two well-known and widely-used questionnaires, contextualize the results based on standard pain-related metrics and compare them to self-reports of general health not specifically tied to pain per se, and maximize the data sample. The standardized assessments used in the present study, ODI and the EQ5D QoL, were chosen for their broader evaluation of a patient’s wellness and function, including reported disability and quality of life, and their ability to evaluate a state-dependent wellness rating, as opposed to assessments aiming to assess trait-like behavior. The open-ended self reports were collected across a select period of time during the clinical trial (October 2021-October 2022), in which participants were asked to respond periodically via text message to open-ended, free-text questions about a variety of pain-related symptoms, and generally about their day. The prompt questions are reported in Supplementary Table 1.

All available data was downloaded and eligible for use in the clustering analysis regardless of which time point it originated in the clinical trial time course (e.g., baseline/enrollment, SCS trial period, follow-up). This allowed us to capture a broad range of clinical information across time in chronic pain, consistent with the way current assessments are applied across the course of the condition. It also allowed us to focus on state- and population-level patterns rather than individual trajectories. This decision was intentional, both to parallel the time-independent nature of standard assessments and to account for the heterogeneous number of observations per participant. A total of 14 questions (Table 2) were analyzed: overall/leg/back pain, mood, sleep hours, sleep quality, alertness, medication use for opioid/over-the-counter/non-opioid pain medication, activity interference due to pain, and activities of daily life). Although these questions were custom and thus non-standard, they were designed to reduce patient burden relative to traditional long-form questionnaires, and elicit fast, consistent responses from patients about pre-established, pain-relevant topics within a digital ecosystem.

Most of questions were answered by the subjects along the clinical study. However, there were some days that subjects did not answer every question on every day. In this case, we used linear and Markov chain methods to impute data based on these criteria: at least 60% of historic data available for the subject and less than 14 consecutive days of missing data. We then used the following criteria: 1) For a given day, the participant had responded to all questions or missing variables were reasonably imputed; 2) Daily averages were calculated in the instances in which participants responded to a question more than once per day; 3) We only considered subjects for which we had more than 10 data points. Notably, after data cleaning and quality control for our clean sample, some subjects were included in the present analysis ultimately had fewer than 10 eligible data points. To ensure that these individuals were not driving the results, we repeated our analyses excluding these individuals, and found only negligible differences in terms of the cluster characteristics compared to the full sample. To produce a reduced set of stable composite scores for each category and reduce autocorrelation across the variables, the data to assess pain, sleep, and medication use were averaged within each modality. Activity scores were calculated where the total number of reported activities of daily living were penalized using the pain interference score (see Table 2 for details). For pain medication, we calculated the mean of the three categories (prescription opioid, prescription non-opioid, and over-the counter) from the participant response, which indicated how much medication was taken relative to their usual amount (none (0), less (1), the same (2), or more (3) medication). All data were then normalized and scaled between 0 and 1 prior to being incorporated into the clustering analysis.

In an additional, exploratory analysis, we analyzed and examined a cluster solution that included the questionnaire data described above in combination with the actigraphy data. For the actigraphy data specifically, we calculated effective mobility^22,36^, a novel metric of physical function based on the duration and intensity of activity. To do this, rates of activity were calculated periodically throughout the day, and assigned to a category ranging from Zone 0 (e.g., resting, or using a mobile device while seated) to Zone 4 (e.g., intense or repetitive motion or vigorous exercise). The same imputation criteria described above was applied for this feature.

Additional study data characteristics, including retention rates, available data for all data types, and slope of reported responses, can be found in the Supplementary Materials (Supplementary Figs. 7–9).

### Validation data preparation

Finally, several steps were taken to prepare the validation data. We extracted standardized assessments of disability and quality of life (ODI, QoL, respectively, mentioned above) with the intention of later comparing them to the state assignments. These were collected less frequently than the daily questionnaires and were administered both in-clinic and using the at-home phone app. For this work, we used the total ODI score and sub- and total scores of EQ5D, including those for activities of daily living (ADL), Mobility, Pain, Mood, Health (VAS) and the total index score, to evaluate and clinically interpret each cluster.

To preprocess the text data, we extracted text responses from open-ended free-text prompts presented to participants (see Table 2). Transformers using RoBERTA^37^ were used to compute semantic similarity between their responses and different topics (Supplementary Table 2) such as “I have more pain” or “I have less pain” as well as other aspects in their life, including mood, sleep quality and time, socialization, stimulator device function and recommendations, job performance, exercise, attentiveness, medication, anxiety, and chores^38^. This was aggregated using principal component analysis (PCA) into a consistency measure and assigned a value, which was interpreted as a text health score associated with each response on a positive to negative health spectrum. This was later used to compare to the assigned state.

### Clustering methods

Clustering was calculated for 1) questionnaire data only and 2) questionnaire data combined with watch-based actigraphy data. This was performed in this order because watch data was unavailable for all individuals on all days, thus limiting sample size yet providing valuable non-subjective insight. We used the k-means clustering algorithm to examine how the participant symptom grouped together across time. K-means was chosen for its unsupervised nature and ability to handle larger sets of data, but several alternative methods were explored to verify convergence (e.g., factor analysis, PCA, hierarchical clustering). The k-means analysis was performed using Euclidean distance exploring solutions for up to k = 10. The optimal k was determined using the majority agreement of multiple standard methods including sum of squares distances, silhouette values, and consensus clustering^39^, a method adapted for our analysis to resample based upon subject number, adding subject-specificity and rigor to standard stability metrics (Supplementary Fig. 1). Consensus clustering in particular also helps to mitigate the impact of repeated measures by reducing overfitting to individual-level idiosyncrasies and enhancing cluster stability. The results of the stability analyses were taken into account along with prior findings and topic expertise.

The cluster solution results were then replicated for consistency across periods of time within the clinical trial, including before and after SCS therapy (Supplementary Fig. 2). For this analysis, the clusters were examined over periods of time defined by the clinical trial and with the intention of maximizing data per time window. Periods of 4 months were chosen for periods of time ranging from 4 months prior to SCS implantation up to 12 months after implantation, and then yearly up to 3 years following implantation. A three-state solution was used to maximize cluster stability; we did not opt for a larger cluster solution due to known instability for k-means in small samples. For each of these time periods, the k = 3 cluster solution was calculated, graphed, and examined. We confirmed that the feature means tracked with expected characteristics – for example, we observed that three months prior to and following SCS implantation showed very high values for pain and medication use, and similarly, the pain levels decreased post-SCS over time. Furthermore, the clusters maintained some consistency over time (see Supplementary Fig. 2), most specifically showing similar feature means and cluster shape for the best and worst states. Additionally, participants with varying response rates (Supplementary Fig. 3), including those that responded at relatively high and low rates, were examined, also using a 3-state solution. Results indicated consistency across these groups. Finally, Transition periods were also reviewed across all states to ensure that the state solution maintained the expected properties of biological systems (Supplementary Fig. 4). Multidimensional scaling (MDS) was used to examine the similarity of the centroid characteristics across two dimensions (Supplementary Fig. 6).

### Clustering validation

The clustering results were then compared to the independent validation data. This allowed us to compare the well-known standard assessments in the validation to the more novel daily questions, given that the daily digital questions were custom and thus non-standard, created with the intent for use in a digital environment. These standard validation assessments were collected in-clinic at baseline, months 1, 3, and 12, and yearly. In the Envision study, participants also responded to the assessments within the phone-based digital ecosystem at more frequent, varying intervals, most often being defined as every 3 weeks. To address the validation, we obtained the standard assessments described above (*e.g*., ODI, QoL) and identified the cluster assigned to a participant within a week of that particular standard assessment. This meant that for these pairs, we used a cutoff of 7 days across the two data points to ensure that they were close enough in time to be clinically related. Thus, if the response date for either item in the pair of metrics was collected outside the one-week window, they were dropped from the analysis. From the state assignments, we extracted the centroid distance for all clusters in the optimal solution. We calculated the Pearson r correlation and *p*-value between the cluster centroids and the standard assessments with which it was paired. Next, we repeated this analysis with the text health score, comparing within-week pairs of the text health score to the state centroids.

In order to cross-check the validation, this analysis was repeated in a smaller sample (118 samples across 76 participants) in the Global Impression of Change^40^ for both physician and patient subscales using the model without actigraphy in order to maximize data availability. While it was found that both of these scales ranked the states in the same way that the ODI did, not all values were found to be significant, likely due to the difference in sample size. These values are reported in Supplementary Table 4.

Finally, to further understand the role of pain in shaping the identified states, we repeated the analysis once more while excluding pain questions from the model. As in the previous models, the resulting 5-state solution produced 5 clusters that were also distributed on an apparent positive-to-negative spectrum (Supplementary Fig. 10a). Correlations with clinical assessments supported this interpretation (Supplementary Fig. 10b). Although the correlation magnitudes (r-values) were slightly weaker than when pain was included, these findings did suggest that non-pain features also contribute meaningfully to well-being in individuals with chronic pain. While additional studies are needed for confirmation, these findings also indicate that a model using similar non-pain features might successfully be applied across different types of chronic pain.

## Supplementary information

Supplement_CP_HealthMetric_NPJDM_Revision_Aug2025

## Data Availability

Restrictions apply to the availability of these data due to expectations of privacy which were outlined at the time of consent. Further, they are used under license for the purposes of the current study. As a result, they are not publicly available. Data may be made available upon reasonable request in accordance with Institutional Review Board and Data User Agreement limitations. Requests can be made by contacting Boston Scientific at Dat.Huynh@bsci.com.

## Code availability

The underlying code for this study is not publicly available for proprietary reasons, but example and pseudocode have been provided in Python and can be found at: https://github.com/jennamr/IBM-pain-states.
