# Supplementary material for: Defining and validating a multidimensional digital metric of health states in chronic back and leg pain
Source: NPJ Digit Med. 2025 Nov 21;8:713. doi: 10.1038/s41746-025-02084-1 (PMC12639008; doi:10.1038/s41746-025-02084-1)
Supplement: Supplementary file 1 — Supplement_CP_HealthMetric_NPJDM_Revision_Aug2025 [file 41746_2025_2084_MOESM1_ESM.pdf]

Supplementary Materials for

Defining and validating a multidimensional digital metric of health states in chronic back and leg pain

Would you like to share any additional information about the importance to you to any of the following factors: overall pain, leg pain, low back pain, sleep time, sleep quality, mobility/daily activities, mood, pain medication(s), or other factors that might be important to you?
Would you like to share any additional information about the trade-offs you indicated you might be willing to make related to the following: overall pain, sleep time, sleep quality, mobility/activity, medications, mood, and/or any other factors that are important to you?
Please tell us more about why you selected your answer to the previous question about how well the recommendations worked for you. For example, I you indicated that you would not want to received recommendations in the future, what (if anything) could be done to change your mind? If you indicated you would want to receive recommendations in the future, please expand on why you would want to, and how the recommendations made you better.
How was your day today? Can you share how the recommendations from the app may have made a difference?
You selected 'Other' in the previous question, can you expand a bit more why?

Supplementary Table 1. Text prompts used in the NLP analysis. Open-ended free text questions were presented to participants, prompting a response in (smartphone-based) text format. These responses were analyzed for semantic similarities between the free-text responses and pre-determined target concepts (e.g., pain, mood, sleep, socialization, activity, etc.) NLP was used to categorize response valence, and a text health score for the text was computed.

**"The recommendation was beneficial"**
**"The recommendation was neutral"**
**"The recommendation was detrimental"**
**"I did implement the recommendation"**
**"I did not implement the recommendation"**
**"I have less pain intensity"**
**"I have the same pain intensity"**
**"I have more pain intensity"**
**"My mood is better"**
**"My mood is worse"**
**"My mood is the same"**
**"My mood is worse"**
**"I am more attentive"**
**"I am less attentive"**
**"I am less anxious"**
**"I am more anxious"**
**"I am socializing more"**
**"I am socializing less"**
**"I am using less medication"**
**"I am using more medication"**
**"I am exercising more"**
**"I am exercising less"**
**"I can do more house chores"**
**"I can do less house chores"**
**"The device is working"**
**"The device is not working"**
**"It is easier to do my job"**
**"It is more difficult to do my job"**
**"I am sleeping more"**
**"I am sleeping less"**
**"I am sleeping better"**
**"I am sleeping worse"**

Supplementary Table 2. Statement comparisons used in the NLP (Natural Language Processing) analysis. Transformers were used with RoBERTA to compute semantic similarity between participants' text responses and the different topics shown in this table. Logical consistency was calculated between the open-form text responses and these statements in order to compute a text health score, in which larger numbers were associated with more positive responses.

Model with NO Effective Mobility			
	R Value	95% CI Low	95% CI High
State A*	-0.23	-0.25	-0.20
State B*	-0.28	-0.31	-0.26
State C *ns*	0.02	-0.01	0.04
State D*	0.15	0.13	0.18
State E*	0.21	0.18	0.23
State (Number)	-0.22	-0.24	-0.19
Overall Pain	-0.14	-0.16	-0.11

Model with Effective Mobility			
	R Value	95% CI Low	95% CI High
State A_M*	-0.28	-0.31	-0.25
State B_M*	-0.12	-0.15	-0.09
State C_M*	-0.15	-0.18	-0.11
State D_M*	0.15	0.12	0.18
State E_M*	0.23	0.20	0.26
State (Number)	-0.22	-0.25	-0.19
Overall Pain	-0.16	-0.19	-0.13

Supplementary Table 3. Comparison Between States and Free-Form Text Responses. Natural Language Implication was used to compute a health summary metric from open-form, free-text responses. A correlation was then calculated between centroid distance from each state, as well as from a total of numbered states (where A = 1; E = 5, etc.) and daily reported overall pain. Asterisk denotes significance at $p < 0.000001$; *ns* = not significant.

	State A	State B	State C	State D	State E
**ODI Total**	**0.34**	**0.22**	**-0.12**	**-0.28**	**-0.51**
**Patient GICr**	**0.35**	**0.25**	-0.03	-0.07	**-0.31**
**Clinician GICr**	**0.24**	0.12	-0.09	-0.10	**-0.28**

Bold denotes significance at p<0.05

Supplementary Table 4. Additional correlation calculation. In order to ensure that the validation analysis could be used across various pain-related measurements, the ranking was checked against the Global Impression of Change (GIC) for both the patient and clinician scales, which included 118 samples in 76 participants. It was found that the ranking was the same as that that was provided by the ODI (1031 samples in 332 participants), but not all values achieved significance, perhaps due to the smaller sample size.

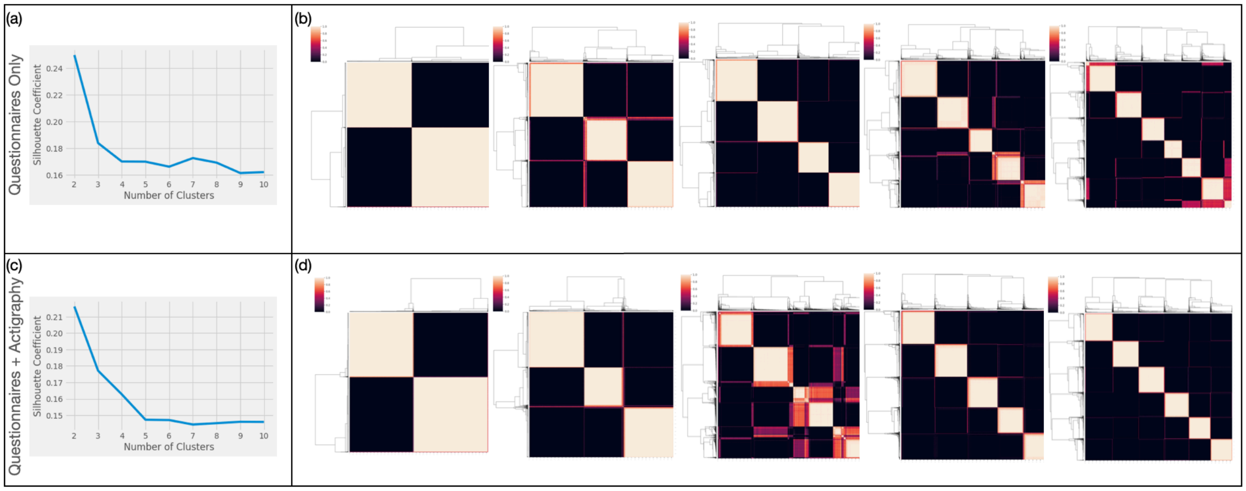

Supplementary Figure 1. Cluster stability analysis. Standard stability measures were calculated using Silhouette Scores up to k=10 (a, c) and consensus clustering up to k=6 (b, d) for solutions without actigraphy (a, b) and with actigraphy (c, d). Relatively stable solutions were observed for both models up to k=3, with stability degrading for k=4 in the model with actigraphy, and in k=6 in the questionnaire-only solution. Additional tests, not shown here, were performed to verify results. Taken collectively, we chose a solution of k=5 in order to maximize number of stable clusters across analyses with and without actigraphy, but to avoid solutions for either analysis that included an unstable solution (k=4, k=6).

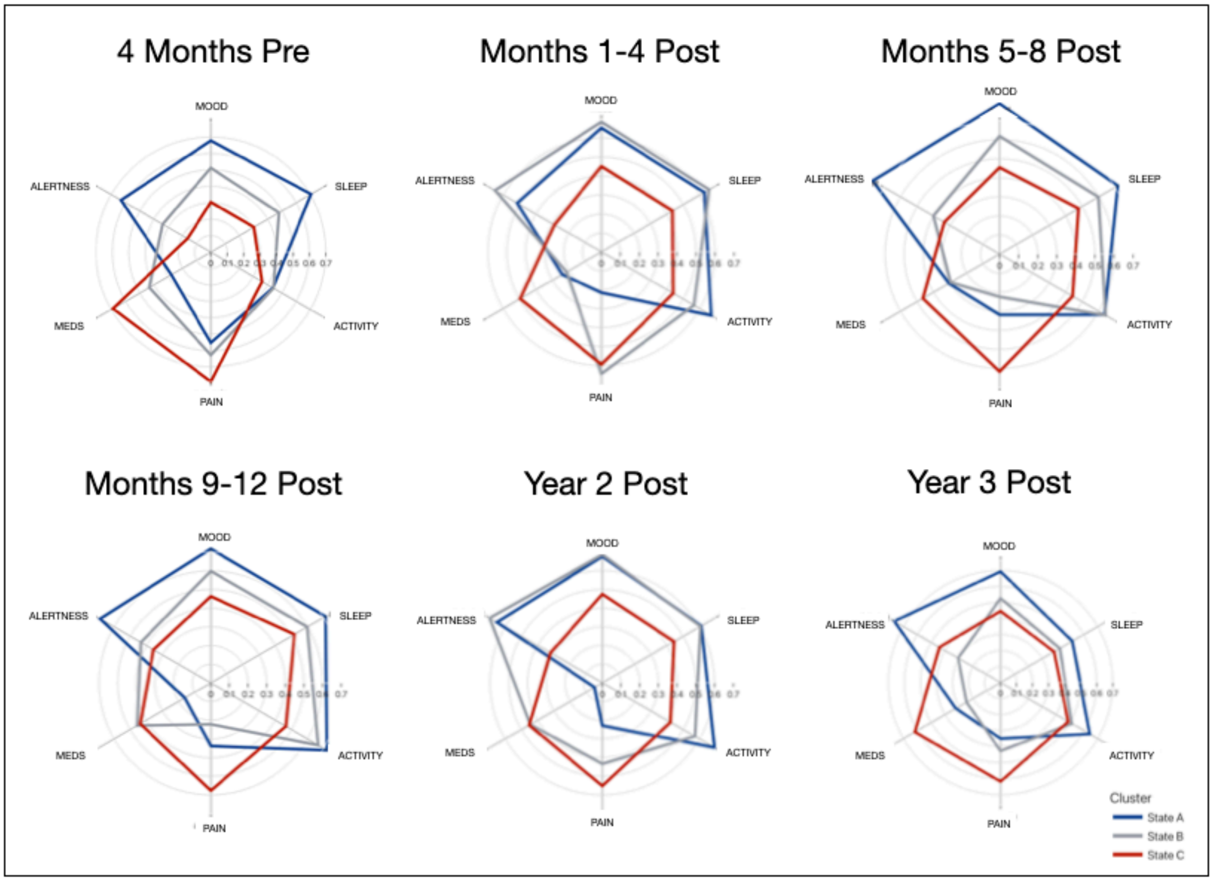

Supplementary Figure 2. Cluster solutions across time. Cluster solutions were recomputed across periods of time designated by predetermined clinical trial events and milestones relative to spinal cord stimulation implantation. Because each computation involved only a selected portion of data relative to the entire sample, k = 3 was used because larger cluster solutions were deemed to have been unstable using these smaller input sample sizes. Here, we show that with some expected differences (e.g., differences in pain and medication use at the beginning and end of study) there is a degree of consistency in the presence of a best, middle, and worst state across time. Note that here, unlike in some other graphs, we show raw (not inverse) values for pain and medication use.

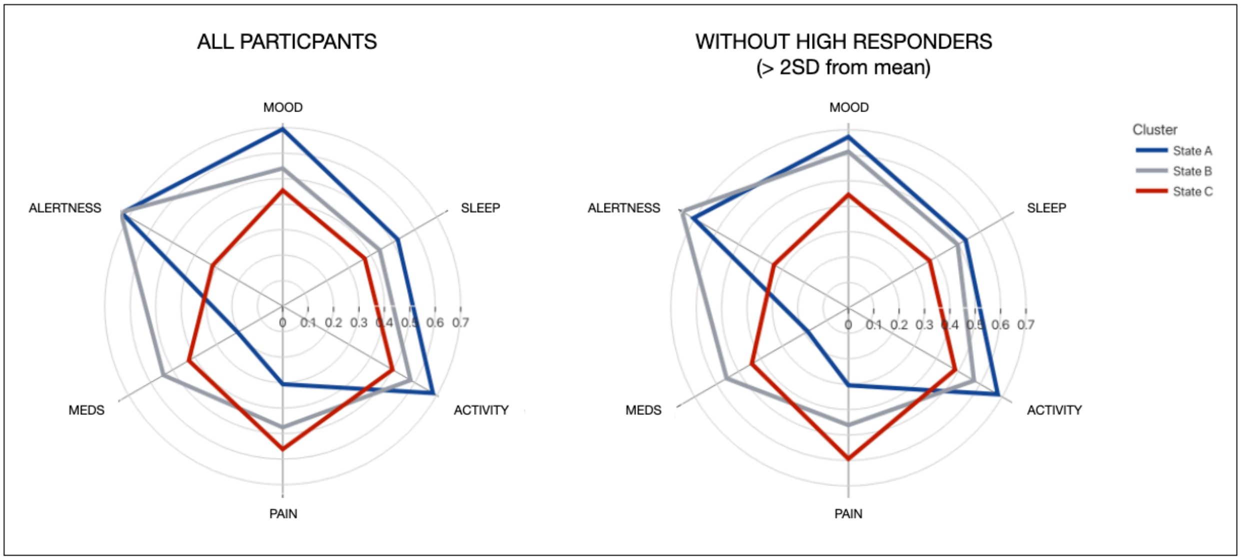

Supplementary Figure 3. Clusters with and without High Responders. Analyses were run first with all participants (left), and then repeated without the participants with a very high response rate (>2 SD above the mean response rate) to determine whether the high responders were driving the effects of the cluster results. As in prior analyses that partitioned the dataset, a solution of k = 3 was used to avoid instability associated with smaller sample sizes. Here, we found that the cluster solutions were similar even when high responders were eliminated from the analysis. Note that here, unlike in some other graphs, we show raw (not inverse) values for pain and medication use.

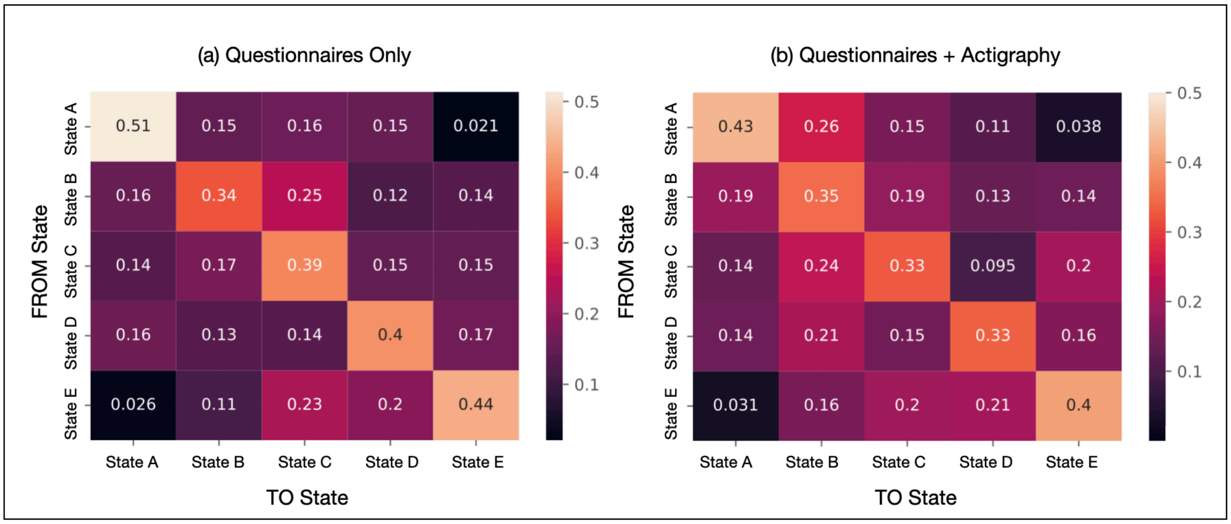

Supplementary Figure 4. **State Transition Probabilities.** Transition probabilities are shown (a) without effective mobility, and (b) with effective mobility. The matrix was examined for expected properties, such as same-state stability, which was observed given the largest transition probabilities were observed along the diagonal. Also expected, we observed that values transitioning between extremes (for instance from State A to E, and vice versa) were very low. Finally, some indication of improvement across time was observed, given that probabilities of moving from E to other states was smaller than moving from A to other states.

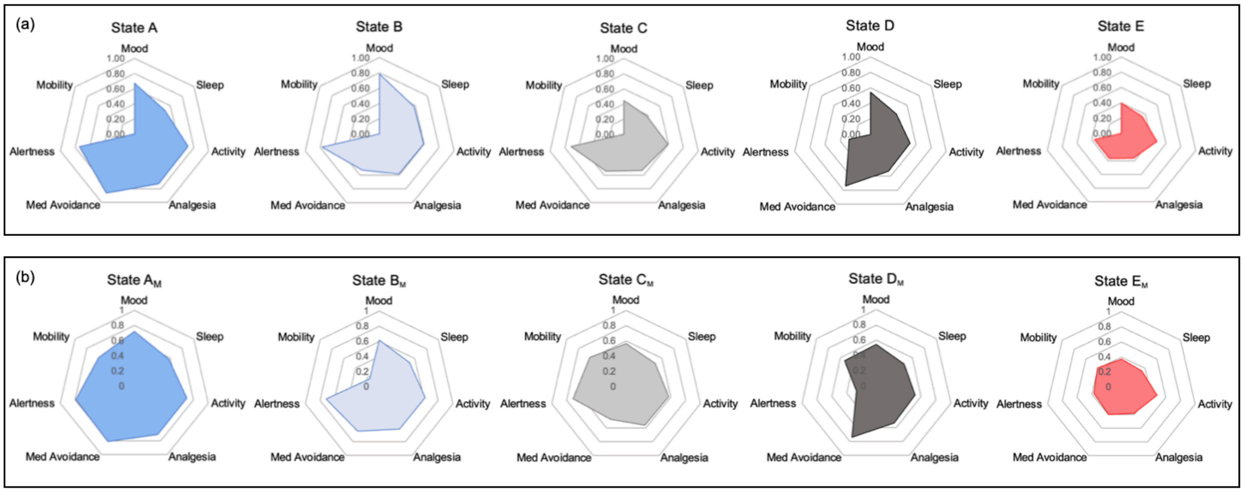

Supplementary Figure 5. K-means results for a 5-cluster solution with visual comparison with and without actigraphy. The clustering results derived from questionnaires alone (above) revealed 5 states that were stratified on a negative-to-positive spectrum (note, inverse values are taken for pain and medication to visualize all feature values on the same good-to-bad scale). The best state (State A) demonstrates a high mean of mood, sleep, activity, and alertness, analgesia (1-reported pain), and medication avoidance (1-medication use). Conversely, the worst state (State E) shows the opposite pattern. These results are shown for data using (a) states A-E resulting from questionnaire data only, and (b) states A_M – E_M resulting from both questionnaire and mobility (actigraphy) data. Unlike in the main text, for (A), a 0-value placeholder has been inserted for mobility so that the models may be directly visually compared.

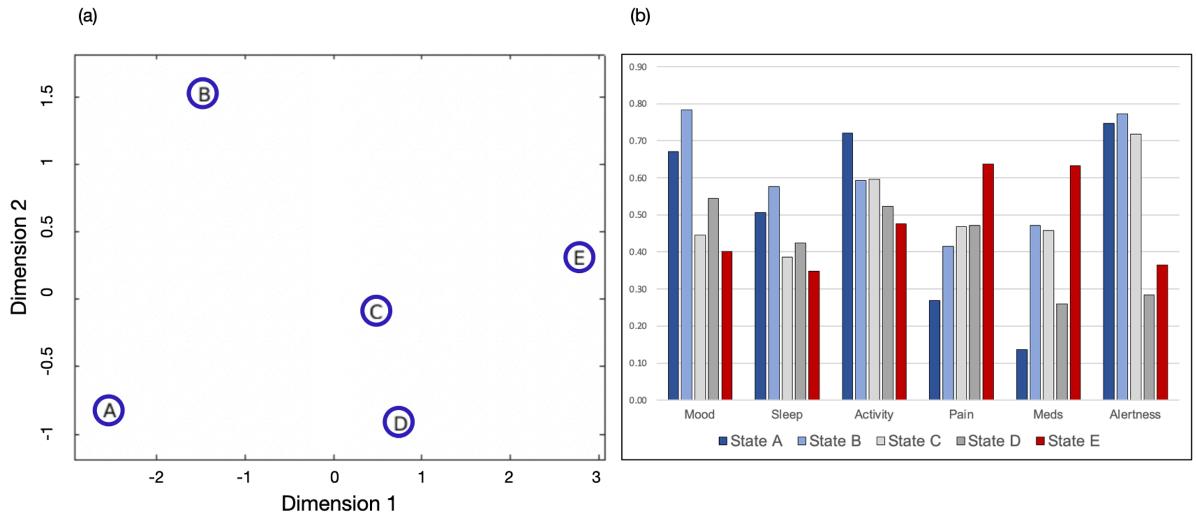

Supplementary Figure 6. Multidimensional scaling (MDS) of state centroids. To further understand the structure of the centroid characteristics, we used a multidimensional scaling representation of the States A-E (a). Here, we observe that Dimension 1 stratifies the states in the same order as did the correlation validation analysis. Dimension 2, however, shows a distinct differentiation, suggesting another variable could underlie the stratification of states. For instance, medication use (b) is a potential candidate that may drive this effect. Note the main text shows this variable in the inverse for visualization purposes; here it is presented to show the relationship to the results of this analysis.

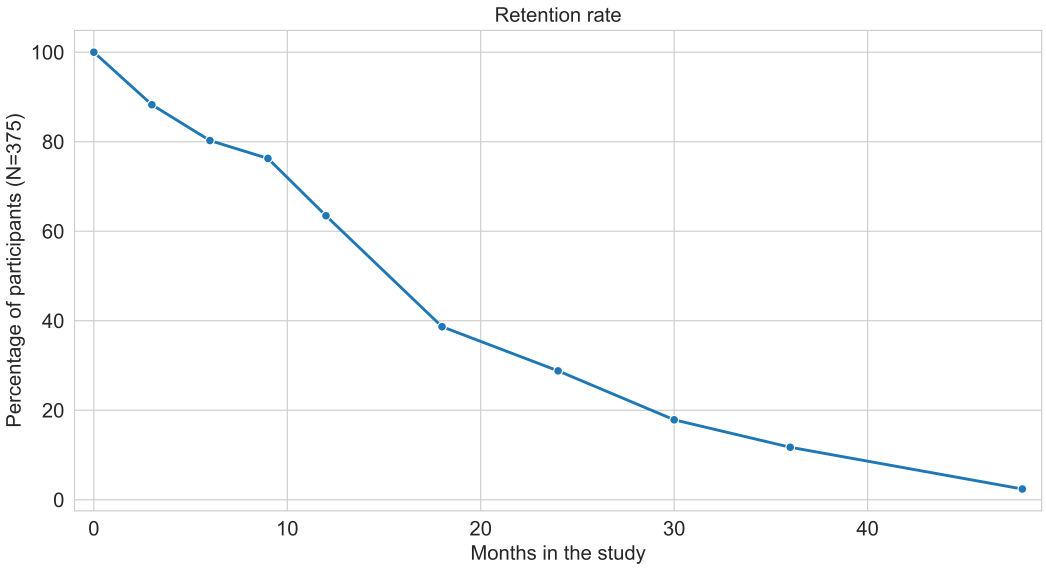

Supplementary Figure 7. Participant Retention Rates. Retention rates were calculated based on individual participant length of time in study prior to completion, or withdrawal for any reason (drop-out, lost-to-follow-up, removal, etc.).

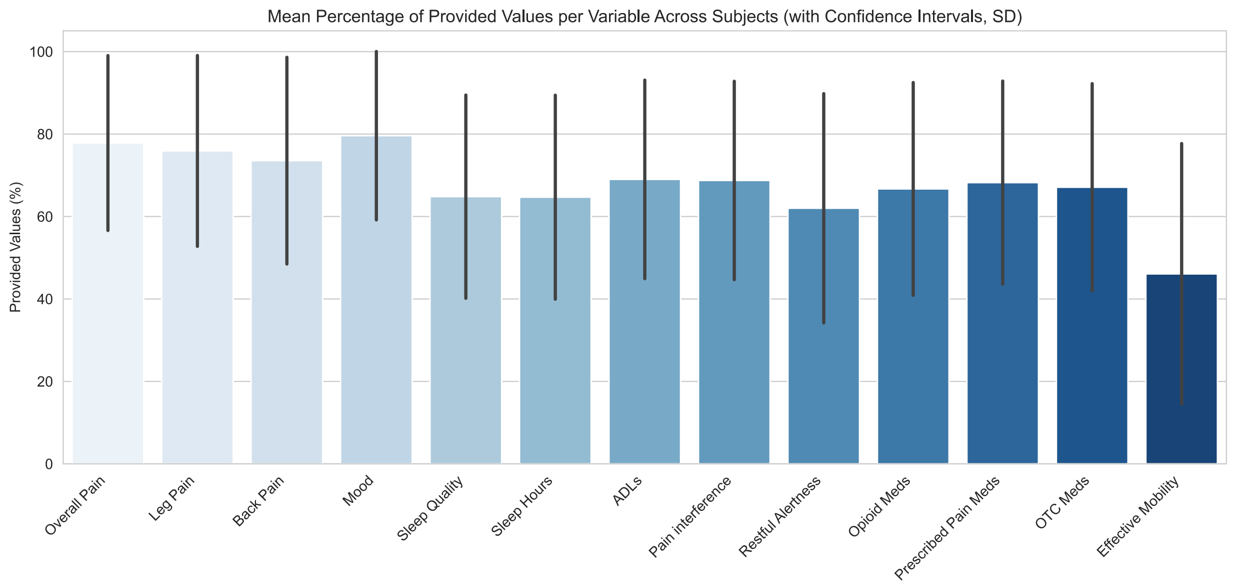

Supplementary Figure 8. Available Data for Each Data Type. For each participant included in the present analysis, mean available data across days was calculated. The bar graph here shows mean available data for each data type, and the standard deviation across participants for all digital data types across the duration of the study. ADLS = activities for daily living; OTC = over-the-counter medications.

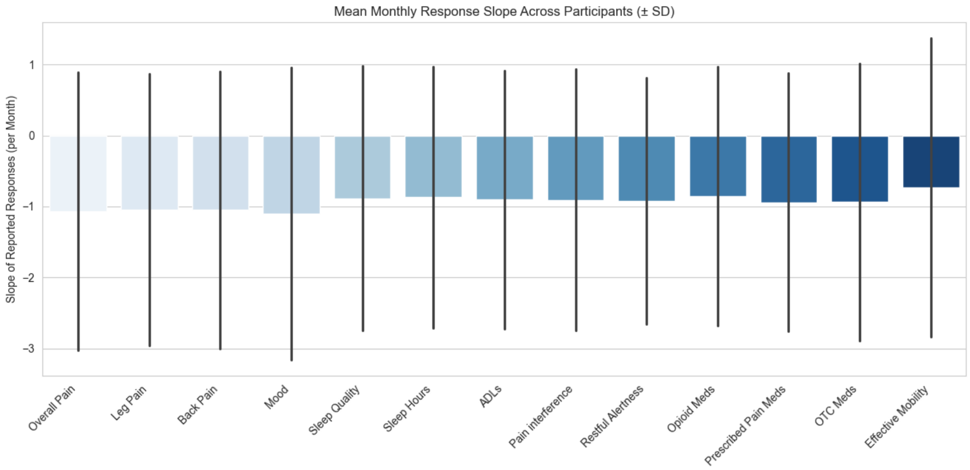

Supplementary Figure 9. Slope of Reported Responses. We calculated the slope of reported responses over time, using one-month windows and participants who had at least 3 months of data. Findings suggest substantial variability between participants, with a negative slope on average as expected in digital health studies. The median amount of data loss was ~1 response per month on average.

(a)

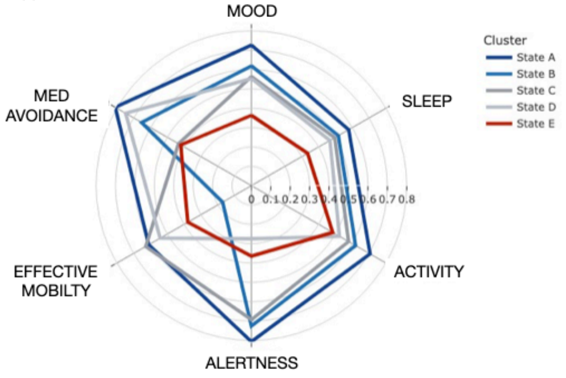

(b)

	State A	State B	State C	State D	State E
ODI Total	0.33	0.09	-0.04	-0.14	-0.42
EQ5D ADL	0.19	0.00	0.01	-0.06	-0.23
EQ5D Mobility	0.20	0.04	-0.04	-0.18	-0.31
EQ5D Pain	0.27	0.12	-0.04	-0.12	-0.32
EQ5D Mood	0.15	0.08	-0.07	-0.04	-0.20
EQ5D Health (VAS)	-0.19	-0.01	0.08	0.05	0.29
EQ5D Index Score	0.29	0.08	-0.05	-0.14	-0.38

Supplementary Figure 10. 5-State Solution Without Pain. To evaluate the contribution of pain to the model, we recalculated the 5-state cluster solution after removing the pain variables. Excluding pain items still produced a 5-state solution distributed across a positive to negative health spectrum (a). Confirming this, the centroids remained correlated with the standard assessments (b), though with smaller r-values than in the model including pain. This suggests that non-pain features contribute meaningfully to well-being in chronic pain.
